# Reviewed article: Rocha WP, Reuter T, Gaspar PC, Leitão FNC, Barille G, Boldrini NAT, Silveira MF, Miranda AE. Prevalence and factors associated with high-risk human papilomavírus infection in women living with HIV/AIDS at a referral center: a cross-sectional study, Vitória, 2021-2022. Epidemol Serv Saude. 2025:34;20240796

**DOI:** 10.1590/S2237-96222025v34e20240796.a

**Published:** 2025-10-10

**Authors:** Guilherme Lamperti Thomazi

**Affiliations:** 1 Aids Healthcare Foundation, Porto Alegre, RS, Brazil Aids Healthcare Foundation Porto Alegre RS Brazil

## Peer review A

## Questionnaire

Does the manuscript contain new and significant information to justify publication? Yes

Does the Abstract (Summary) clearly and accurately describe the content of the article? Yes

Is the problem significant and concisely stated? Yes

Are the methods described comprehensively? Yes

Are the interpretations and conclusions justified by the results? Yes

Is adequate reference made to other work in the field? Yes

## Manuscript Structure

Length of article is: Adequate

Number of tables is: Adequate

Number of figures is: Adequate

Please state any conflict(s) of interest that you have in relation to the review of this paper. None

Interest: Good 

Quality: Good

Originality: Good

Overall: Good 

Recommendation: Minor Revision

## Comments

Gostaria de parabenizar pela temática do artigo, trago algumas considerações e sugestões a partir da leitura do trabalho.

1. Sugiro a mudança de “mulheres com HIV para mulheres com HIV/AIDS”. Homogeneizar os termos, em alguns momentos fala “HIV” em outros “HIV/AIDS”;

2. Acho importante especificar, brevemente, que se tratam de mulheres cisgêneras ou existiriam homens trans?

3. Sugiro marcar os subtitulos de alguma forma (como Local do estudo), está com a mesma fonte, tamanho e formato que o texto corrido, dificultando a leitura;

4. Qual o motivo da escolha do intervalo etário de 18 a 64 anos?

5. Não ficou claro se as entrevistas semiestruturadas foram feitas presencialmente ou não, pois falam de formulário online.

6. Motivo da escolha de indetectavel com CV inferior a 20, sendo que diversos manuais colocam inferior a 40?

7. “Um total de 207 mulheres (85,9%)” esse 85,9% se refere a amostra estimada?

8. Fala em alguns paragrafos das dificuldades de acesso de mulheres vivendo com HIV/AIDS na discussão mas não cita algum artigo que fale sobre. Sugiro os artigos: https://journals.sagepub.com/doi/abs/10.1177/09564624241263614 “Case-control study on challenges in loss of follow-up care and the limitations in the reach of HIV policies for women”; https://www.scielo.br/j/spmj/a/tW4nVtS5pyPYSkdm7YWVfFC/ “Adherence to antiretroviral therapy among women living with HIV/AIDS in the interior of the Brazilian state of Pará: cross-sectional study.”; e https://pubmed.ncbi.nlm.nih.gov/29376409/ “Social and structural determinants of HIV treatment and care among black women living with HIV infection: a systematic review”.

9. Nessa mesma esteira, sugiro repensar a divisão de raça/cor. O IBGE divide em brancos, pretos, pardos, amarelos e indígenas. Sendo a junção de pretos + pardos nomeada negros. Acredito que após isso deva ser discutido a questão do racismo no acesso a saúde, tendo em vista a importância de tal variavel.

10. Não entendi a categoria “Diagnóstico IST dado pelo médico” tendo em vista que diversos outros profissionais podem dar o diagnóstico de ISTs, a maioria sendo da enfermagem.

11. Adicionar outras fragilidades e limitações do artigo na discussão, como: e as mulheres com HIV/AIDS que não estão nos serviços ou mesmo sabem de seu diagnóstico?

12. Acredito que a discussão sobre barreiras de acesso possa enriquecer a discussão, importante levar em consideração que muitas mulheres não acessam os serviços pois elas são responsáveis por cuidar da família, muitas vezes se deixando de lado, como sugere o trabalho de Gabriela Tizianel sugerido acima.

